# Intravaginal nonablative radiofrequency in the treatment of genitourinary syndrome of menopause symptoms: a single-arm pilot study

**DOI:** 10.1186/s12905-021-01518-8

**Published:** 2021-10-30

**Authors:** Cintia Pinheiro, Teresa Costa, Raira Amorim de Jesus, Raquel Campos, Rosa Brim, Alcina Teles, Andrea Vilas Boas, Patrícia Lordêlo

**Affiliations:** 1 Pelvic Floor Care Center (CAAP), Bahiana School of Medicine and Public Health, Av Dom João VI, 275 – Brotas, Salvador, Bahia 40290000 Brazil; 2 Pelvic Floor Center Care, Av. ACM, 1034, Itaigara, Salvador, Bahia 41825-906 Brazil; 3 Bahia State University, Rua Silveira Martins, 2555, Cabula, Salvador, Bahia 41150-000 Brazil

**Keywords:** Genitourinary syndrome of menopause, Menopause, Radiofrequency

## Abstract

**Background:**

Genitourinary syndrome of menopause (GSM) involves vaginal dryness (VD), pain during sexual activity (SAPain), vaginal itching (VI), burning, pain, and symptoms in the urinary organs. Non-ablative radiofrequency (RF) is a type of current with electromagnetic waves with a thermal effect that generates an acute inflammatory process with consequent neocolagenesis and neoelastogenesis. We aimed to describe the clinical response to VD, SAPain, vaginal laxity (VL), VI, burning sensation, pain in the vaginal opening, urinary incontinence, sexual dysfunction, cytological changes, and adverse effects of non-ablative RF in patients with GSM.

**Methods:**

This single-arm pilot study included 11 women diagnosed with GSM with established menopause. Patients with hormone replacement initiation for six months, who used a pacemaker, or had metals in the pelvic region, were excluded. Subjective measures (numeric rating scale of symptoms, Vaginal Health Index-VHI) and objective measures (vaginal maturation index-VMI, vaginal pH, sexual function by the FSFI, and urinary function by the ICIQ-SF) were used. A Likert scale measures the degree of satisfaction with the treatment. Five sessions of monopolar non-ablative RF (41°C) were performed with an interval of one week between each application. The entire evaluation was performed before treatment (T0), one month (T1), and three months (T2) after treatment. Adverse effects were assessed during treatment and at T1 and T2.

**Results:**

The symptoms and/or signs were reduced after treatment in most patients (T1/T2, respectively): VD 90.9%/81.8%, SAPain 83.3%/66.7, VL 100%/100%, VI 100%/100%, burning 75%/87.5%, pain 75%/75%, and VHI 90.9%/81.9%. Most patients did not show changes in VMI (54.5%) and pH (63.6%) at T1, but there was an improvement in VMI in most patients (54.5%) at T2. Nine patients were satisfied, and two were very satisfied at T1. The treatment was well tolerated, and no adverse effects were observed. There was an improvement in sexual function (72.7%) and urinary function (66.7% in T1 and 83.3% in T2).

**Conclusion:**

Intravaginal RF reduced the clinical symptoms of GSM in most patients, especially during T1, and women reported satisfaction with treatment. The technique showed no adverse effects, and there were positive effects on sexual and urinary function.

*Trial registration* This research was registered at clinicaltrial.gov (NCT03506594) and complete registration date was posted on April 24, 2018.

## Background

Genitourinary syndrome of menopause (GSM) is traditionally defined as a set of signs and symptoms due to altered estrogen production, both physiologically or as a result of a therapeutic approach. It involves physical and sensory changes in the external and internal genitalia and lower urinary tract region, such as loss of collagen and elastin, altered smooth muscle cell function, reduction in the number of blood vessels, and an increase in connective tissue, leading to epithelial thinning, decreased blood flow, and reduced elasticity [1]. Women may have some or all the signs and symptoms. The most common symptoms are vaginal dryness (VD), pain during sexual activity (SAPain), and urinary incontinence (UI) [2]. It is estimated that 10%- 45% of these women live with some discomfort due to GSM. However, only 25% seek treatment, and symptoms are unlikely to improve spontaneously [2–4].

GSM treatment aims to alleviate symptoms and reverse atrophic anatomical changes. Hormonal therapy is the current gold standard treatment that can be administered systemically or locally [1, 5, 6]. However, there are contraindications such as history of breast cancer, coronary artery disease, previous venous thromboembolic event or stroke, and adverse effects, such as vaginal bleeding, endometrial hyperplasia, breast pain, and perineal pain, which limit its use [3, 6, 7]. Approximately 23% of women using hormonal therapy reported that they felt incomplete or no relief of vaginal/vulvar symptoms and 36% of patients noted that the vagina did not restore their natural state [8].

With the contraindications and limitations of standard therapy for GSM, a search for new therapeutic options for GSM management is needed. Radiofrequency (RF), a new alternative technique for GSM [9], is a high-frequency current used for therapeutic purposes, based on the mechanism of heat production by conversion, that is, ionic and molecular mobilization, favoring oxygenation, nutrition, and vasodilation of tissues [10]. The heating of the tissues also promotes the denaturation of collagen with a subsequent contraction of its fibers, retraction of fibrous septa, and activation of fibroblasts. Neocolagenization, neoelastogenesis, and reorganization of collagen fibers may occur, resulting in tissue remodeling [10–12].

Based on the knowledge of the physiological responses of the tissues submitted to RF and on the results of its use on the treatment of genitourinary signs and symptoms related to GSM, this research aimed to describe the clinical response (VD, SAPain, vaginal laxity (VL), vaginal itching (VI), burning sensation, pain in the vaginal opening, UI, and sexual dysfunction), cytological changes, and adverse effects of non-ablative RF in patients with GSM.

## Materials and methods

### Study design

This was a single-arm pilot study preceding a randomized controlled trial (RCT) in progress, followed the precepts of the Declaration of Helsinki, with the approval of the Ethics and Research Committee of the Bahiana School of Medicine and Health (EBMSP) with CAAE 72147317.9.0000.5544 on September 5, 2017, and registered at clinicaltrial.gov (NCT03506594) and complete registration date (first date posted) on April 24, 2018. Written informed consent was obtained from all patients.

Adult women with established menopause (at least 12 months after their last period and/or bilateral oophorectomy) and who had complaints of at least one of the symptoms of GSM (VD, SAPain, VL, VI, burning sensation, and pain in the vaginal opening) participated in the study. The women were referred by gynecology services, and the service took place at the teaching outpatient clinic of the Physiotherapy Clinic at EBMSP. For inclusion in the study, they should have a vaginal pH of ≥ 5 and vaginal cytology from the last 12 months, or three previous normal tests, without any malignancy and/or atypia. We excluded patients with hormone replacement initiation for six months, who used a pacemaker, or had metals in the pelvic region, hemophiliacs, using vasodilators and/or anticoagulants, and those with chronic neurological degenerative diseases and/or diagnosis of current vaginal infection.

### Assessment procedures

Initially, we administered a basic anamnesis questionnaire for collecting sociodemographic and clinical data. Each participant subjectively assessed their symptoms (VD, SAPain, VL, VI, burning sensation, and pain in the vaginal introitus) using the **Numeric Rating Scale (NRS)**, which consists of a scale from 0 to 10 points, with 0 indicating no symptoms and 10 indicating as many symptoms as possible.

The physical examination was to assess the **vaginal health index (VHI)**, which consists of a graduated scale from 1 to 5 for each item (vaginal elasticity, fluid volume, pH, epithelium integrity, and humidity). Vaginal elasticity varies between 1 (no elasticity) and 5 (excellent elasticity), assessed through mucosal distention upon palpation and in the placement of the speculum. Fluid volume, assessed at inspection, varies between 1 (no secretion) and 5 (normal secretion) (white flocculent). Epithelium integrity varies between 1 (petechiae already detected on inspection) and 5 (tissue not friable and normal mucosa). Moisture varies between 1 (no moisture detected in the inspection and presence of an inflamed mucosa) and up to 5 (normal humidity). The pH was quantified using a pH indicator strip between 0 and 14 (MColorpHast™—pH-indicator strips) which was placed directly on the right lateral vaginal wall for one minute, giving 1 point for the pH of 6.1, and 5 for the pH ≤ 4.6, in which the last one was considered normal. The sum of all items represents the vaginal health score, with 25 representing the best vaginal health [13].

The **Vaginal Maturation Index (VMI)** was evaluated from a vaginal secretion collection, in the middle third of the vaginal canal, fixed in absolute alcohol and sent to a laboratory, in which a biomedical doctor performed the percentage counting of the parabasal (P), intermediate (I), and superficial (S) cells, characterizing the vaginal epithelium in hypotrophic (I > S), normotrophic (I = S), hypertrophic (I < S), mild atrophic (I > P), moderate atrophic (I >  = P) and marked atrophic (I < P). The sum of the three cell types totals 100% and is presented as follows: P/I/S [14].

Participants answered the **Female Sexual Function Index (FSFI)** [15] questionnaire to objectively assess sexual function, gathering responses in six different domains: desire, arousal, lubrication, orgasm, satisfaction, and discomfort/pain. The cut-off point of ≤ 26.5 was considered for sexual dysfunction and the increase in the score was considered an improvement. The **Sexual Quotient-Female Version (QS-F)** assessed women’s sexual activity. This questionnaire was developed and validated in 2006, specifically for the Brazilian population [16]. Through ten self-answering questions, the QS-F assesses all phases of the sexual response cycle with a total index ranging from 0 to 100. Higher values indicate better sexual performance and satisfaction.

To assess the impact of UI on quality of life and characterize urinary loss, we used the **International Consultation on Incontinence Questionnaire-Short Form (ICIQ-SF)** [17], composed of five questions that assess the frequency, severity, and impact of UI, in addition to a set of eight items of self-diagnosis related to UI situations experienced by patients. The maximum sum of the response values indicates a score of 21 points, referring to the high impact of UI on an individual's life.

At the end of the treatment, the participants were asked about their degree of satisfaction with the treatment using a five-point Likert scale, which classified the patient's level of satisfaction as 1 (very dissatisfied), 2 (dissatisfied), 3 (unchanged), 4 (satisfied), 5 (very satisfied) [18].

To assess the clinical response, we considered an improvement when there was a decrease in values in self-reported symptoms, verified by the NRS; the increase in the value of the VHI; the decrease in parabasal cells (deep) and/or increase in superficial cells evaluated by VMI, a decrease in vaginal pH, an increase in FSFI and QS-F scores, a decrease in the sum of the ICIQ-SF questions, and improved satisfaction according to the five-point Likert scale.

We used all outcome measures before, one month, and three months after the end of treatment, respectively, times T0, T1, and T2, performed by the same initial evaluators.

To test safety, we considered existing adverse effects if they had erythema, ulcers, fistulas, burns, blisters, bleeding, and/or pain. They were evaluated during each application at T1, T2, or at any contact by the patients’ self-reports. We considered it to be at risk if it had one or more of these effects. If an adverse effect occurs, the patient was referred for evaluation and treatment by the team’s gynecologist. The RF treatment would be interrupted, and the data are presented in the results.

### Therapeutic procedure

**Fig. 1:**
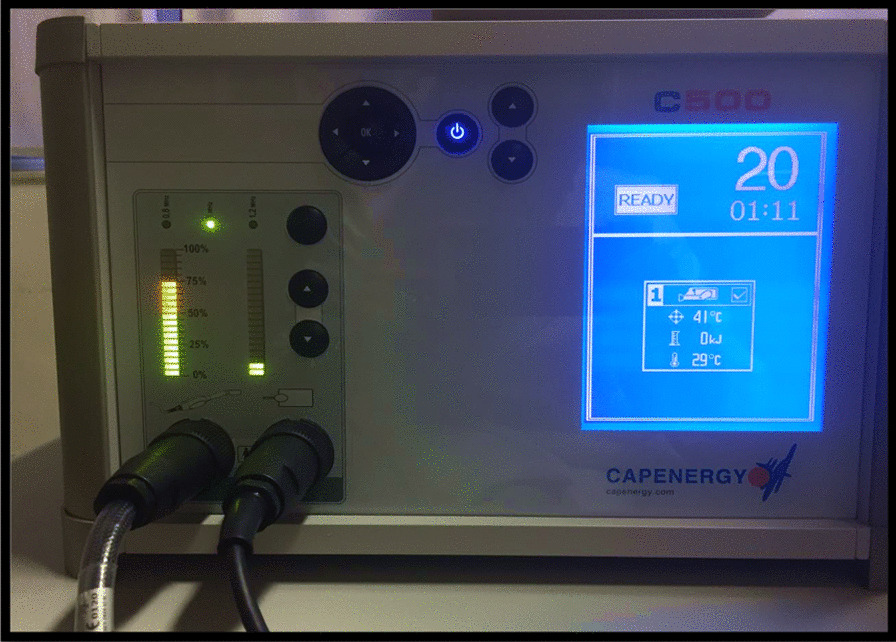
Radiofrequency device (Capenergy®)

**Fig. 2:**
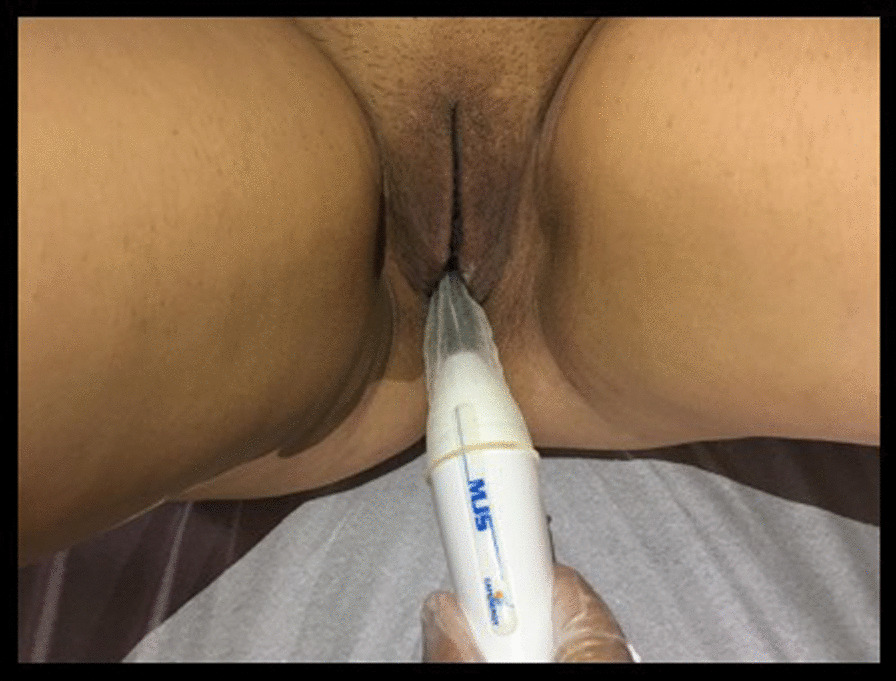
Radiofrequency application (Capenergy®) intracavitary with semicircular movements

RF was used in the form of capacitive electrical transfer, monopolar configuration (Capenergy® device, model C500), which has two electrodes: an intracavitary active electrode placed in the vagina with a non-lubricated condom and water-soluble gel and another electrode, dispersive, positioned in the lumbosacral region (Fig. 1). For the application, the participants were placed in the supine position with the lower limbs abducted and the knees bent. The temperature was set at 41°C, with a frequency of 1 MHz and power of 75 kJ. When the established temperature was reached, the physiotherapist maintained it for 2 min with semicircular movements on the anterior wall of the vagina. The movement and time on the posterior vaginal wall was repeated, totaling 4 min of after reaching the established temperature (Fig. 2). Each patient underwent five RF sessions, with an interval of seven days between them.

### Data analysis

For the elaboration of the database, we used the software Statistical Package for Social Sciences (SPSS), version 14.0 for Windows. The results were reported descriptively in the text or through tables and graphs; categorical variables are expressed as absolute and percentage values—n (%), and continuous variables with normal distribution in mean and standard deviation (± SD), and those with asymmetric distribution in the median and interquartile range (IQ).

## Results

### Sociodemographic and clinical characteristics

The sample consisted of 11 patients with GSM, with an average age of 59.6 ± 3.93 years, with data collected from October 2017 to August 2018. Table 1 presents the sociodemographic and clinical characteristics of the study population. The main characteristic was VD, as they all had this symptom. Menopause duration ranged from two to 17 years, with a median of 14 years (6–15).

**Table 1:** Sociodemographic and clinical characteristics of 11 patients with GSM

Sociodemographic characteristics	
	*Mean ± SD*
Age	59.6 ± 3.93
Education level	n (%)
Complete higher education	5 (45.5)
Complete high school	4 (36.4)
Complete Elementary school	1 (9.10)
Illiterate	1 (9.10)
Marital status	
Married	4 (36.4)
Divorced/separated	3 (27.3)
Single	2 (18.2)
Widow	2 (18.2)
Self-reported skin color	
Brown	7 (63.6)
Black	2 (18.2)
White	2 (18.2)

Clinical characteristics	n (%)
Body mass index	
Low weight	1 (9.10)
Normal	3 (27.3)
Overweight	6 (54.5)
Obesity	1 (9.10)
Hormone replacement	5 (45.5)
Gynecological surgeries	9 (81.8)
Constipation	4 (36.4)
Number of deliveries	
Nulliparous	1 (9.10)
Primiparous	1 (9.10)
Multiparous	9 (81.8)
Type of delivery	
Vaginal	5 (45.5)
Cesarean	5 (45.5)
Symptoms of GSM	
Vaginal dryness	11 (100)
Pain during sexual activity	6 (54.5)
Vaginal laxity	6 (54.5)
Vaginal itching	6 (54.5)
Vaginal burning	8 (72.7)
Vaginal pain	4 (36.4)
Urinary incontinence	6 (54.5)
Vaginal intercourse/penetration/coitous	
No	6 (54.5)
Sexual dysfunction	8 (72.7)
Vaginal color	
Whitish	6 (54.5)
Normal	5 (45.5)

### Symptoms of GSM

We observed the clinical improvement of GSM in the NRS scores of the symptoms of VD, SAPain, VL, VI, burning, and vaginal pain, as shown in Table 2, especially in T1.

**Table 2:** Numerical Rating Scale of the symptoms of Genitourinary Syndrome of Menopause before treatment, 1 month and 3 months after 05 radiofrequency sessions

Patient	Pre-treatment						1 month						3 months					
	VD	SAPain	VL	VI	B	Pain	VD	SAPain	VL	VI	B	Pain	VD	SAPain	VL	VI	B	Pain
1	10	10	0	5	10	0	3	5	0	0	0	0	7	7	0	5	6	7
2	9	8	0	0	0	0	3	0	0	3	0	0	6	0	0	4	0	0
3	10	0	8	10	9	10	7	0	6	9	10	6	5	0	2	9	10	2
4	5	0	10	7	5	0	0	0	8	0	0	0	0	0	7	0	2	0
5	8	2	5	0	0	2	4	0	1	0	1	0	8	1	4	0	0	0
6	7	7	0	0	0	0	1	1	1	0	0	0	1	0	0	0	0	1
7	10	0	3	2	4	0	2	0	0	0	0	1	1	0	0	0	0	0
8	10	0	2	0	4	0	1	0	0	0	2	0	1	8	0	0	2	0
9	7	7	0	6	5	7	7	7	2	5	5	7	7	0	3	4	4	7
10	9	8	5	5	8	8	1	0	0	0	0	0	1	0	0	0	0	0
11	10	0	0	0	9	0	5	0	2	0	4	0	1	0	1	0	1	0

*VD* vaginal dryness, *SAPain *pain during sexual activity, *VL* vaginal laxity, *VI* vaginal itching, *B* burning

### Vaginal Health Index (VHI)

Ten patients (90.9%) showed an increase in the VHI score in T1, which represents an improvement, and one worsened (patient five). Regarding pre-treatment, in T2, nine patients (81.8%) had an increase in VHI, while two (18.2%) had a reduction in this index during the initial value (Table 3).

**Table 3:** Vaginal Health Index, Vaginal Cytology, Vaginal pH, FSFI, Sexual Quotient, and ICIQ in 11 patients with GSM, pre-treatment, 1 month, and 3 months after 05 radiofrequency sessions

Patient	PRE						1 month						3 months					
	VHI	pH	VMI %B/I/S	FSFI	QS	ICIQ	VHI	pH	VMI %B/I/S	FSFI	QS	ICIQ	VHI	pH	VMI %B/I/S	FSFI	QS	ICIQ
1	14	5.5	0/90/10	8.5	14	0	25	4.0	0/90/10	14.7	28	0	21	5.0	10/90/0	11.7	30	0
2	12	6.5	40/60/0	3.0	22	0	23	5.0	6/90/4	2.4	24	0	19	5.0	5/95/0	5.0	22	0
3	18	5.0	0/95/5	3.4	16	14	21	5.0	0/80/20	6.2	44	13	22	5.0	0/95/5	6.2	44	11
4	18	6.0	100/0/0	3.0	18	3	20	6.0	20/70/10	7.0	28	7	17	6.0	90/10/0	4.8	44	0
5	18	6.5	100/0/0	24.3	64	0	15	7.0	100/0/0	27.4	72	0	14	6.5	90/10/0	24.4	64	0
6	14	5.0	0/95/5	4.4	66	8	22	5.0	0/95/5	4.0	74	11	20	5.0	0/95/5	3.8	74	9
7	11	7.0	90/10/0	2.6	20	11	13	6.0	90/10/0	20.4	76	0	15	6.0	95/5/0	4.7	18	0
8	17	6.0	100/0/0	23.8	50	12	18	6.0	95/5/0	27.9	60	7	23	5.0	5/90/5	28.2	62	4
9	16	5.0	0/95/5	3.2	24	0	17	5.0	0/90/10	3.2	46	11	19	5.0	0/68/32	10.6	34	13
10	16	5.0	0/95/5	22.7	70	6	20	5.0	0/95/5	31.8	88	4	22	5.0	0/90/10	31.8	90	4
11	22	5.0	0/90/10	4.4	86	0	24	5.0	0/90/10	4.8	60	0	23	5.0	0/90/10	3.6	76	0

*VHI* Vaginal Health Index, *pH* vaginal pH, *%B/I/S* % basal/intermediate/superficial cells, *FSFI* Female Sexual Function Index, *QS* sexual quotient—female version, *ICIQ* International Consultation on Incontinence Questionnaire—Short Form, *VMI* Vaginal Maturation Index

### Vaginal pH

In T1, most patients (7–63.6%) did not change, three (27.3%) showed a decrease in pH, representing improvement, and one (9.1%) showed an increase. In T2, there was also a predominance of maintaining baseline values (Table 3).

### VMI

The cytological analysis showed that six patients (54.5%) remained unchanged in the cell count, and five (45.5%) showed an improvement in T1, with two patients of the latter changing their category from moderate or severe atrophic to mild atrophic. In T2, six patients (54.5%) showed improvement compared to the beginning, three (27.3%) remained at the beginning, and two (18.2%) worsened. One patient maintained the category of severe atrophy, and the other went from hypotrophic to severe atrophy (Table 3).

### Sexual function

In the FSFI analysis, all patients experienced sexual dysfunction during pre-treatment. There was an increase in the total index in nine patients (81.8%) in T1, with three patients without sexual dysfunction. In T2, eight patients (72.7%) had improved compared to the beginning, but half had a decrease compared to T1, and two continued without sexual dysfunction. According to the **QS-F,** seven patients (63.6%) had sexual dysfunction at T0. Ten (90.9%) and seven (63.6%) patients improved their scores at T1 and T2 respectively, compared to the beginning. We observed worsening of the two periods evaluated in Patient 11 (Table 3).

### Urinary symptoms

In this study, six patients had pre-treatment urinary complaints. When assessing the impact of UI on quality of life (QoL) using the ICIQ-SF questionnaire, we found a decrease in the score in four patients (66.7%) in T1, one of whom had no symptoms; two (33.3%) increased the score and one who had no symptoms started to complain (patient nine). In T2, almost all patients (five—83.3%) had an improvement in the beginning, in which two had their symptoms disappeared, and two had a higher score than the initial one (Table 3).

### Satisfaction with treatment

**Fig. 3:**
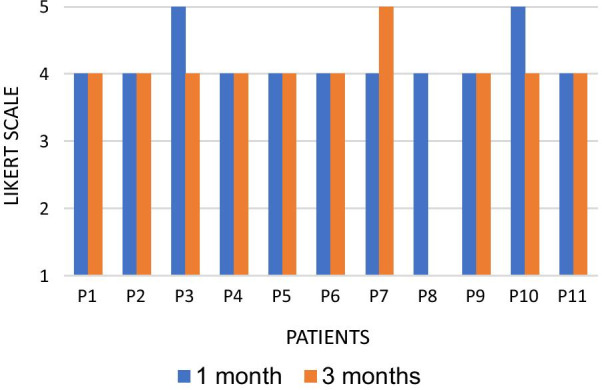
Satisfaction with the treatment of 11 patients with GSM, 1 month and 3 months after 05 radiofrequency sessions

Regarding treatment satisfaction, nine patients (81.8%) reported being satisfied and two (18.2%) were very satisfied at T1. In T2, nine patients (81.8%) remained satisfied, one (9.1%) very satisfied, and one (9.1%) very dissatisfied (Fig. 3). The patient who reported being very dissatisfied with the reassessment during T2 showed improvement in all other parameters evaluated and reported being satisfied after one month.

### Adverse effects

We did not observe erythema, ulcers, fistulas, blisters, burns, bleeding, and/or pain at any time during treatment or follow-up. The treatment was well tolerated. One patient reported a little discomfort in the lower abdominal region during the first two sessions, which ceased in the third application.

## Discussion

We found an improvement in genitourinary symptoms such as VD, SAPain, VL, VI, burning sensation, pain in the vaginal opening, UI, and sexual dysfunction in most patients, especially in T1, in this pilot study that used non-ablative RF in postmenopausal women with GSM. The application of RF was considered safe because only one light adverse effect in one patient was found up to T2. To our knowledge, this is the first study to assess symptoms of GSM associated with cytological analysis and patient satisfaction after using non-drug and non-ablative treatment with intracavitary monopolar RF.

VD was the main symptom reported by patients in this study and showed an important improvement when assessed by both self-report and by VHI. Based on the histological changes observed in previous studies, the process of neocolagenesis and neoelastogenesis that occurs after exposure to controlled RF thermal energy in the vaginal tissue restores most vaginal functions such as secretion, absorption, elasticity, lubrication, and tissue consistency, which are decreased in GSM [9, 19, 20]. This hypothesis is supported by previous knowledge that this high-frequency current induces collisions and movements between atoms and molecules, resulting in energy transfer to the tissue in the form of heat and a consequent controlled increase in temperature, promoting an increase in the arterial circulation, and vasodilation, and improving tissue oxygenation [21].

VD can generate or contribute to SAPain, just as abstinence from intimate relationships is involved in the decline of lubrication, often forming a cycle. Vaginal trophism is fundamental for a comfortable sexual intercourse, which depends on the lubrication promoted by vasodilation of the lamina propria and the vaginal epithelium [22, 23]. In this study, a patient who did not have SAPain in the pre-treatment started to report this symptom in T2. An assumption is that an increase in the frequency of sexual activity may favor the appearance of this symptom. To confirm this hypothesis, an investigation of sexual frequency before and after treatment must be included. We are developing an RCT in which this variable has been included and, therefore, we may soon have this information. The increase in sensory perception by neurogenesis can occur after using RF [20], which can lead to a greater perception of the vagina; therefore, some patients may start to report these symptoms. However, further morphometric investigations of neuronal analysis are necessary.

Other studies have also found positive results for SAPain. Alinsod (2015) studied RF with controlled temperature (TTCRF) with intra- and extra-vaginal application in six menopausal women and 10 in the peri-menopausal period with SAPain symptoms, demonstrating the safety and beneficial effects of the treatment [24]. An RCT with 20 postmenopausal women applied three sessions of intra- and extra-vaginal TTCRF (ThermiVa) once a month with reduced VD and SAPain [25].

RF has also been widely used to improve collagen levels. Its diathermic effects cause the collagen denaturation. As the temperature rises, some of the cross-links are broken, causing the triple helix to unwind. Thus, there is a consequent activation of fibroblasts, with subsequent neocolagenesis, neoelastogenesis, and tissue remodeling [26]. Coad et al. (2013) evaluated the histological effect of non-ablative RF on the vaginal introitus of sheep before, after seven, 30, and 90 days. There was a significant increase in the activation of the submucosa fibroblasts and an increase in collagen compared to the control group [27]. In a histological study in multiparous sows, a significant progressive increase in the amount of elastin and collagen in the vaginal mucosa was observed. The treatment consisted of weekly intravaginal radiofrequency administrations for three weeks, with follow-up at both one week and one month after treatment [28]. The evaluation of the vaginal wall, performed by ultrasound, showed an increase in thickness, but it was not statistically significant [28]. In our study, the NRS was used to grade VL, and all patients noted improvement. Three patients who did not have the complaint initially reported mild intensity in the reassessments, which can also be justified by the increase in sensory perception after treatment. Assessing the symptoms of GSM is challenging because of its subjective nature. The development of a specific score with a cut-off point for the quantification of these symptoms and clinical improvement may be of great relevance to better evaluate these patients and verify the treatment effect.

Previous studies have obtained positive results with the use of monopolar RF with cryogenic surface cooling in pre-menopausal women with a history of at least one vaginal delivery and complaints of vulvovaginal atrophy/symptoms of GSM or VL [26, 29]. Alinsod (2015) used the TTCRF in 23 pre-menopausal women with VL, with improvement on a seven-point scale, called the vaginal laxity questionnaire (VLQ) [30]. Krychman et al. (2017) carried out a multi-center study with 189 premenopausal women complaining of VL during sexual intercourse with significant improvements in self-report and sexual function by FSFI [29].

Symptoms such as VI, burning, and pain are common complaints in GSM. Hypoestrogenia results in reduced number of epithelial layers and vessels, thinning of smooth muscles [31], and an increase in nociceptive sensory afferents [32]. In addition, the increase in tissue friction caused by the decrease in trophism and moisture causes greater mucosal fragility, contributing even more to the condition [22, 33]. In this study, there was an important improvement in these symptoms. This clinical improvement is supported by the thermal effect of RF, which significantly affects the tissue layers. As a consequence of local peripheral vasodilation and increased blood flow, there is an improvement in trophism, oxygenation, cellular metabolism, and lubrication [10, 34]. High-frequency thermal therapy seems to act through the effects of analgesia, but the mechanisms by which RF controls pain are still unclear, seemingly involving the transduction of C fiber signals [35].

The analysis of vaginal cytology through the VMI and the measurement of pH are well-used measures to establish diagnostic parameters of GSM [36, 37], but have not yet been analyzed in RF research in GSM. Brizzolara et al. (1999) carried out a study in 70 postmenopausal women determining a specific vaginal pH range that correlates with high levels of parabasal cells in the VMI, defined as at least 20%, and found a correlation between these two objective measures [38]. In this study, pH values ≥ 6.0 were compatible with a greater number of parabasal cells (≥ 20%). However, these results showed that there was no pattern of clinical/objective findings with the symptoms reported by the patients. VMI and pH, unlike NRS, remained similar in most patients (six-54.6% and seven-63.3%, respectively) in T1. In T2, most patients showed an improvement in VMI (54.6%) and in pH (36.4%). Vaginal cytology, in part, has been inconsistent with clinical findings [36]. A smaller-scale and more sensitive tape is recommended to detect minor variations.

Two patients presented with worsening VMI, which may have happened at random, because it is a small sample or due to the aging process. This change could be better controlled by an RCT. In the literature, there is no evaluation made of the agreement between evaluators of the VMI, which is a dependent evaluation instrument.

GSM has adverse effects on sexual function and general well-being. The FSFI is an instrument used worldwide to assess sexual function. In this study, it improved in most of the samples in T1 (81.8%) and T2 (72.7%). Patients were also evaluated using QS-F, a questionnaire specifically developed for the Brazilian population [16]. In terms of the total score, we observed an increase in QS-F in most of the participants. The promising results and the last position statements published by the European Society of Sexual Medicine [39] stimulated our group to carry out an RCT of RF in the treatment of signs and symptoms of GSM that is in progress. Using the non-ablative RF technique, Lordelo et al. (2016) carried out an RCT with 43 women dissatisfied with the appearance of their genitalia. They applied RF to the external genitalia, with an improvement in sexual function by 3.51 points in the group treated in the evaluation by FSFI [11].

RF is considered one of the most innovative non-surgical modalities for treating UI and VL [40]. In addition to modifying the trophism of the vaginal canal, it also targets the urethral mucosa and seems to improve not only the symptoms of GSM but also those of UI. This change could be better controlled by an RCT. In our study, although the type of UI has not been classified, 66.7% and 83.8% of the patients improved urinary symptoms at T1 and T2, respectively. Lalji & Lozanova (2017) in a pilot study, conducted three treatment sessions with monopolar RF, intra- and extra-cavitary, in 27 women with stress urinary incontinence (SUI). They found that 96.3% decreased the frequency of urinary loss by at least one level, and 59.3% reported a decrease in the amount of loss [41]. Another study with 10 patients with SUI showed improvement in the pad test one month after treatment with monopolar non-ablative RF in the urethral meatus [42]. Despite different outcome measures and application forms, RF therapy appears to be a good alternative for the treatment of SUI. Histological studies have observed a reduction in collagen in the urethral walls in the event of loss of urethral support and/or internal sphincter dysfunction [43], which supports the use of RF in this dysfunction.

Treatment satisfaction was assessed using a five-point Likert Scale, with most patients reporting satisfaction with treatment. This was reinforced by the decrease in symptoms recorded in that study. On the other hand, we observed that although most of the outcome measures have improved, the indication of patient satisfaction was greater, showing that the degree of satisfaction do not always correspond to the clinical results. Thus, satisfaction is not only linked to the therapeutic result, but also to the level of expectations of the people involved. It is important to consider the Hawthorne effect, which states that when individuals believe they are experiencing a form of treatment, they are more likely to respond and be satisfied with therapeutic responses [44]. In this sense, we also justified carrying out an RCT to better assess this issue.

Our safety analysis showed that the technique was well tolerated by patients and no side effects were observed or reported. In one patient, only mild discomfort was noted in the first two sessions. Also in the study by Lalij and Lozanova (2017), using intravaginal and extracavitary radiofrequency, no side effects were reported [41]. In this study, no description about possible side-effects was given either. The parameters of this study were also different from ours (e.g.: we measured temperature; had five treatment sessions instead of three; applied RF laterolateral instead of longitudinal; and we included a follow-up after three months). Because it was not entirely clear to what extent the different therapeutic parameters would influence safety, we decided to include a safety assessment in our research. We therefore chose to initially conduct a single-arm pilot study with patients presenting GSM. And we only moved on to a Phase 2 trial with a control group and sample calculation [45] once the therapeutic protocol was found to be safe and if there was a therapeutic response. In a safety study conducted in an animal model in treated sows, additional aspects were evaluated, such as the presence of edema, histological changes in the composition of the vaginal wall, change in urination frequency, erythema, and antalgic position, and no side effects were found [28]. A long-term effect that was not evaluated in our study and, to our knowledge, in any other study in the literature, is the possibility that non-ablative radiofrequency enhances the development of tumor cells. A biological effect takes place in the treated area as the electromagnetic field causes a temperature increase [10], and an increased blood circulation. The possibility of cell multiplication is hypothesized, including malignant cells [46]. Therefore, as a safety inclusion, patients had to undergo a vaginal cytology test and show a normal outcome, prior to treatment. For many years the literature has described the effect of thermotherapy (including radiofrequency), which, at a temperature of 41°C or more, and if maintained for at least 30 min, brings a possible therapeutic effect against cancer cells by reducing cellular DNA and RNA synthesis and respiratory depression [47]. The application of radiofrequency in the present study took place at a temperature of 41ºC, but the total treatment time was lower than the recommended time to treat cancer cells, and for this reason it may be important to perform a new long-term cytopathological evaluation to confirm the safety of the treatment technique.

Although some of the patients continued to show improvement in their symptoms in T2, some symptoms were accentuated in that period. Studying the frequency of reapplication after the end of treatment to maintain clinical improvement is essential in future studies, especially since we already have evidence from histological studies (in animals) that show that the peak of RF action occurs within 21 days after its application [10].

## Strengths and limitations

This is a pioneer research on the application of intracavitary non-ablative radiofrequency (RF), in women with Genitourinary Syndrome of Menopause (GSM). The study combines clinical symptoms of GSM, including sexual and urinary functions, with cytological results, patient satisfaction and treatment outcomes.

As it is a non-medicated pharmacological approach, applied locally, it opens up a new field for the treatment of this syndrome. In addition to being a new field for physiotherapy professionals.

As limitations of the study, we found a short follow-up time, the use of independent symptom outcome measures (such as sexual and urinary function questionnaires) for symptoms associated with a syndrome, the use of evaluator dependent indices (VHI and VMI) and a subjective index (VHI).

Based on these limitations, we understand the need to create a specific rating scale for GSM symptoms, intra- and inter-rating comparisons for rater-dependent scales for longer follow-up period and, since intracavitary non-ablative RF has been proven safe, the realization of an RCT.

## Conclusions

The symptoms of VD, SAPain, VL, VI, burning sensation, pain in the vaginal opening, UI, and sexual dysfunction of GSM with non-ablative RF showed clinical improvement in most of the patients, with improvement in the self-report of the symptoms and the VHI, especially at T1. The cytological analysis, through the VMI, remained unchanged in most evaluations in T1, but there was a greater improvement in T2. There was no change in vaginal pH in most patients after RF treatment. There was no adverse effect in the 11 patients evaluated, which is considered a safe and well-tolerated technique, and patients reported satisfaction with treatment.

## Data Availability

The data is available if requested by the corresponding author.

## Abbreviations

B: Burning
EBMSP: Bahiana School of Medicine and Health
FSFI: Female Sexual Function Index
GSM: Genitourinary syndrome of menopause
ICIQ-SF: International consultation on incontinence questionnaire—short Form
IQ: Interquartile range
NRS: Numeric Rating Scale
QS-F: Sexual quotient—female version
RF: Radiofrequency
SAPain: Pain during sexual activity
SD: Standard deviation
SUI: Stress urinary incontinence
TTCRF: Radiofrequency with controlled temperature
UI: Urinary incontinence
VD: Vaginal dryness
VHI: Vaginal Health Index
VI: Vaginal itchy/pruritus
VL: Vaginal laxity
VLQ: Vaginal laxity questionnaire
VMI: Vaginal Maturation Index
